# Supplementary material for: The Evolution and Characterization of the RNA Interference Pathways in Lophotrochozoa
Source: Genome Biol Evol. 2024 May 7;16(5):evae098. doi: 10.1093/gbe/evae098 (PMC11114477; doi:10.1093/gbe/evae098)
Supplement: evae098_Supplementary_Data [file evae098_supplementary_data.zip › Supplementary_materials.zip!/selected_miRNAs/NoGe_selected_miRNAs/novel_miRNA_families_pdf_files/NMRB01000212.1_Notospermus_geniculatus_strain_Ushimado_scaffold212,_whole_genome_shotgun_sequence_7288.pdf]

Provisional ID	:	NMRB01000212.1_Notospermus_geniculatus_strain_Ushimado_scaffold212,_whole_genome_shotgun_sequence_7288
Score total	:	930.5
Score for star read(s)	:	3.9
Score for read counts	:	921.9
Score for mfe	:	1.7
Score for randfold	:	0
Score for cons. seed	:	3
Total read count	:	1820
Mature read count	:	1813
Loop read count	:	0
Star read count	:	7

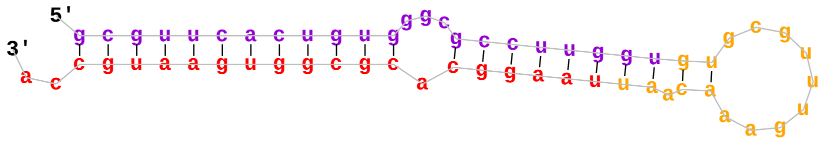

Genome coordinates: NMRB01000212.1 41950-42010

pre-miRNA sequence:
GCGTTCACTGTGGGCGCCTTGGTGCGTTCACTGTGGGCGCCTTGGTGTGCGTTTGAAACAATTAAGGCACGCGGTGAATGCCA

Mature sequence: TAAGGCACGCGGTGAATGCCA

BLAST results for the pre-miRNA sequence against the *Lineus longissimus* genome assembly:

**Scaffold**	**Start**	**Stop**	**Identity**	**e-value**
OU342995.1 1	7123803	7123862	98.333	1.33e-22

```
Nge_novel_miRNA        GCGTTCACTGTGGGCGCCTTGGTGTGCGTTTGAAACAATTAAGGCACGCGGTGAATGCCA
Llo_genome             GCGTTCACTGTGGGCGCCTTGGTGTGCGTTAGAAACAATTAAGGCACGCGGTGAATGCCA
```
